# Editorial: Involvements of TRP Channels, Oxidative Stress and Apoptosis in Neurodegenerative Diseases

**DOI:** 10.3389/fphys.2021.649230

**Published:** 2021-03-11

**Authors:** Nady Braidy, Tarik Smani, Mustafa Naziroglu

**Affiliations:** ^1^Centre for Healthy Brain Ageing, School of Psychiatry, University of New South Wales, Sydney, NSW, Australia; ^2^Biomedical Research Networking Center on Cardiovascular Diseases (CIBER-CV), Instituto de Salud Carlos III (ISCIII), Madrid, Spain; ^3^Department of Medical Physiology and Biophysics, Institute of Biomedicine of Seville, University of Seville, Seville, Spain; ^4^Neuroscience Research Center, Suleyman Demirel University, Isparta, Turkey; ^5^Drug Discovery and Development Research Group in Neuroscience, BSN Health, Analysis and Innovation, Goller Bolgesi Teknokenti, Suleyman Demirel University, Isparta, Turkey

**Keywords:** TRP, oxidative stress, apoptosis, neurodegeneration, aging

The oxygen free radicals generated during metabolism can cause cumulative oxidative damage to nucleic acids, lipids, and protein, resulting in structural degeneration, apoptosis, functional decline, and age-related degenerative diseases involving the cardiovascular, endocrine, neurologic, immune, respiratory, gastrointestinal, and reproductive systems. The transient receptor potential (TRP) protein superfamily is composed of several cation-permeable channels that are widely distributed in mammalian cells. TRP channels can be divided into six subfamilies that are dependent on their sequence identity. These channels play a crucial role in the regulation of oxidative stress and should be considered as likely targets for the treatment of age-related neurodegenerative diseases associated with chronic oxidative stress, decreases in metabolic regulation, and cell viability.

At least three subfamilies of TRP channels are associated with oxidative stress. These include the TRPV subfamily (characterized by chemical, mechanical, and physical stimuli); the TRPC subfamily (characterized by receptor operated calcium entry channels); and the TRPM subfamily (with roles in cell proliferation and death).

The articles in this Research Topic review current thinking with regards to the role of TRP channels in oxidative stress, aging, and neurodegenerative diseases, and highlight the involvement of these channels in the pathobiology of selected neurodegenerative diseases including Alzheimer's disease and Parkinson's disease. Therapeutic strategies that modulate the activation of specific TRP channels may be beneficial for attenuating cellular damage due to oxidative stress in neurological disorders.

The first article by Duitama et al. provides a broad perspective of the role of TRP-dependent mechanism(s), which can mediate pain sensation in neurodegenerative diseases such as Alzheimer's and Parkinson's diseases. It discusses the therapeutic approaches available to palliate pain and neurodegenerative symptoms throughout the regulation of these channels. TRP channels are postulated to be involved in the pathobiology of neurodegenerative diseases and pain nociception through modulation of intracellular Ca2+ signaling, oxidative stress, and the production and release of inflammatory mediators.

The second article by Hong et al. provides an overview of the role of TRP channels as potential targets for neurodegenerative diseases. The review provides an up-to-date summary of the involvement of TRP channels in neurological disorders and discusses recent work on the development of therapeutic candidates for neurodegenerative disorders targeting TRP channels. As the structures of TRP channels are beginning to be elucidated using cryo-electron microscopy, TRP channel antagonists are beginning to be developed to mitigate symptoms in neurodegenerative disorders.

Some TRP channels can be activated downstream of NMDA receptor activation and contribute to reduced synaptic transmission, excitotoxicity, and cell death. The original research article by Doğan et al. examined the interaction between the purinergic receptors (PzX7Rs) and the NMDA receptor in a rat model for absence epilepsy. The findings of this study demonstrate that P2X7Rs play an independent role in the formation of absence seizures. More specifically, treatment with a P2X7Rs agonist lowered the antioxidant activity of the NMDA receptor antagonist memantine whereas the agonist of P2X7Rs lowered the anticonvulsant action of memantine, suggesting a partial interaction between P2X7 and NMDA receptors with potential implications for TRP channels in absence epilepsy. Ru et al. showed that tea polyphenols can attenuate methamphetamine-induced neuronal cell loss by protecting against apoptosis and DNA damage in PC12 cells. Furthermore, ubiquitination appears to be regulated to some extent by TRP channels. The systematic review by Momtaz et al. highlights the involvement of the ubiquitin-proteosome pathway in neurodegenerative diseases and how natural products can interfere with this complex regulatory system at various stages of the disease.

It is well-established that TRPV1 is involved in oxidative stress-induced pain and neuronal injury, associated with neuropathy reported in neurodegenerative diseases and glaucomatous optic neuropathy. An original research article by McGrady et al. provides renewed insight into the role of TRPV1 on optic nerve axon excitability in an animal model for glaucoma. The study found that in the absence of TRPV1, energy demand following intraocular pressure-related stress is increased, leading to alterations to axon transport and maintain optimal voltage-dependent axon signaling. Therefore, in glaucoma, TRPV1 may modulate the expression of voltage-gated sodium channels in neurons exposed to stress to maintain axonal excitability and preserve energy resources.

The TRPV1 channel has also been proposed to act as a steroid receptor to protect tissues against oxidant stress. Ramirez-Barrantes et al. demonstrated that TRPV1 is necessary for 17β-estradiol to improve metabolic function in vulnerable cells. As well, 17β-estradiol but not 17α-estradiol increases the effect of TRPV1 single channel activity leading to increased open probability. The protective effects of 17β-estradiol were found to be independent of estrogen receptor pathway activation, membrane started and stereospecific. These findings suggest that TRPV1 is a 17β-estradiol-activated ionotropic membrane receptor coupling that can influence mitochondrial function and cell viability.

The TRPCs serve as a redox-sensitive ion channel that can play a prominent role in neurodegeneration. Maria-Ferreira et al. review the role of TRPCs in the pathogenesis of Parkinson's disease. The review discusses the role of TRPCs in the various biochemical and molecular processes associated with the pathobiology of Parkinson's disease that consequently led to increased oxidative stress, impaired dopaminergic signaling, and apoptosis.

The TRPM family member TRPM2 has several physiologic isoforms that are present in a variety of cell types and respond to oxidant stress, pro-inflammatory mediators such as TNFα, and β-amyloid peptide. The perspective article by Wang et al. discusses the important role of TRPM2 in Alzheimer's diseases, citing recent studies using divergent cell systems and techniques including overexpression, channel depletion or inhibition, and calcium chelation. The review also provides a causative relationship between exposure to particular matters, TRPM2 channel activation, Alzheimer's disease risk, and age-related brain damage. Therapeutic strategies targeting the TRPM2 channel represent a potential strategy for lowering the risk of exposure to particular matters in Alzheimer's disease.

The mini review article by Santoni et al. provides an overview of the role of endosome/lysosome Ca^2+^ permeable channels known as mucolipins (TRPML) in the regulation of calcium signaling associated with oxidative stress induced oxidative stress. TRPMLs represent a key candidate for the treatment of several neurodegenerative diseases, including Alzheimer's disease, Parkinson's disease, ALS, mucolipidosis type IV, and Neimann-Pick disease.

TRPM7 has been strongly implicated in the regulation of intracellular Ca^2+^ influx and anoxic neuronal cell death. In the last article, Sun et al. demonstrated in a neuroblastoma cell line that treatment with the β-adrenergic receptor (β-AR) agonist isoproterenol could enhance Mg^2+^ influx and cell survival in the presence of the neurotoxin 1-methyl-4-phenyl-1,2,3,6-tetrahydropyridine (MPTP), and potentiate TRPM7 channel activation that leads to an increase in intracellular Mg^2+^ levels. The effect of isoproterenol was reversed following the addition of 2APB, a known TRPM7 channel antagonist. Moreover, TRPM7 expression and function neurotoxins were inhibited following exposure to neurotoxins. These findings suggest a positive role for β-AR in activating TRPM7 channels, modulating Mg^2+^ homeostasis, and promoting the survival of SH-SY5Y cells following exposure to potent neurotoxins.

The sum of the articles adds to our recent work in the area of TRP channel signaling and neurodegenerative diseases. The articles in this special issue provide a summary of the multiple roles of TRP channels in the pathogenesis of neurodegenerative disorders and provide emerging evidence for TRP channels as a target for the development of therapeutic agents to improve neurological dysfunction.

## Author Contributions

NB wrote the manuscript. TS and MN edited the manuscript. All authors reviewed and approved the final manuscript.

## Conflict of Interest

The authors declare that the research was conducted in the absence of any commercial or financial relationships that could be construed as a potential conflict of interest.

